# Participant understanding of informed consent in a multidisease community-based health screening and biobank platform in rural South Africa

**DOI:** 10.1093/inthealth/ihaa072

**Published:** 2020-11-09

**Authors:** Nothando Ngwenya, Manono Luthuli, Resign Gunda, Ntombizonke A Gumede, Oluwafemi Adeagbo, Busisiwe Nkosi, Dickman Gareta, Olivier Koole, Mark Siedner, Emily B Wong, Janet Seeley, on behalf of the Vukuzazi team, Ashmika Surujdeen, Hlolisile Khumalo, Ngcebo Mhlongo, Sanah Bucibo, Sibahle Gumbi, Lindani Mthembu, Seneme Mchunu, Mkhwanazi Phakamani, Mkhwanazi Anele, Mkhwanazi Ntombiyenhlanhla, Myeni Rose, Zikhali Mandlakayise, Mfeka Fezeka, Gumede Hlobisile, Mbonambi Nozipho, Ngubane Hloniphile, Simelane Thokozani, Ndlovu Bongumenzi, Ntimbane Talente, Mbuyisa Mbali, Mkhize Xolani, Sibiya Melusi, Ntombiyenkosi Ntombela, Mandisi Dlamini, Thengokwakhe Nkosi, Sibusiso Mkhwanazi, Mthombeni Skhumbuzo, Chonco Hlobisile, Dlamini Hlengiwe, Mlambo Doctar, Mzimela Nonhlanhla, Buthelezi Zinhle, Steto Mpumelelo, Mhlongo Sibusiso, Magwaza Bongani, Nsibande Siyabonga, Zondi Nombuyiselo, Buthelezi Khanyisani, Nsibande Sibusiso, Nonceba Mfeka, Ayanda Zungu, Hlobisile Gumede, Nonhlanhla Mfekayi, Smangaliso Zulu, Mzamo Buthelezi, Mkhwanazi Senzeni, Mlungisi Dube, Welcome Petros Mthembu, Sphiwe Clement Mthembu, Zinhle Mthembu, Bhengu Thokozani, Sandile Mthembu, Phumelele Mthethwa, Zamashandu Mbatha

**Affiliations:** Africa Health Research Institute, KwaZulu-Natal, South Africa; Division of Infection and Immunity, University College London, London, UK; School of Nursing and Public Health, College of Health Sciences, University of KwaZulu-Natal, KwaZulu-Natal, South Africa; Africa Health Research Institute, KwaZulu-Natal, South Africa; Africa Health Research Institute, KwaZulu-Natal, South Africa; Division of Infection and Immunity, University College London, London, UK; School of Nursing and Public Health, College of Health Sciences, University of KwaZulu-Natal, KwaZulu-Natal, South Africa; Africa Health Research Institute, KwaZulu-Natal, South Africa; Africa Health Research Institute, KwaZulu-Natal, South Africa; Division of Infection and Immunity, University College London, London, UK; Department of Sociology, University of Johannesburg, Johannesburg, South Africa; Africa Health Research Institute, KwaZulu-Natal, South Africa; Division of Infection and Immunity, University College London, London, UK; Africa Health Research Institute, KwaZulu-Natal, South Africa; Africa Health Research Institute, KwaZulu-Natal, South Africa; Global Health and Development Department, London School of Hygiene and Tropical Medicine, London, UK; Africa Health Research Institute, KwaZulu-Natal, South Africa; Division of Infectious Diseases, Massachusetts General Hospital, Boston, MA, USA; Africa Health Research Institute, KwaZulu-Natal, South Africa; Division of Infection and Immunity, University College London, London, UK; Division of Infectious Diseases, Massachusetts General Hospital, Boston, MA, USA; Africa Health Research Institute, KwaZulu-Natal, South Africa; Global Health and Development Department, London School of Hygiene and Tropical Medicine, London, UK

**Keywords:** demographic surveillance, ethics, informed consent, South Africa

## Abstract

**Background:**

In low- and middle-income settings, obtaining informed consent for biobanking may be complicated by socio-economic vulnerability and context-specific power dynamics. We explored participants experiences and perceptions of the research objectives in a community-based multidisease screening and biospecimen collection platform in rural KwaZulu-Natal, South Africa.

**Methods:**

We undertook semi-structured in-depth interviews to assess participant understanding of the informed consent, research objectives and motivation for participation.

**Results:**

Thirty-nine people participated (individuals who participated in screening/biospecimen collection and those who did not and members of the research team). Some participants said they understood the information shared with them. Some said they participated due to the perceived benefits of the reimbursement and convenience of free healthcare. Most who did not participate said it was due to logistical rather than ethical concerns. None of the participants recalled aspects of biobanking and genetics from the consent process.

**Conclusions:**

Although most people understood the study objectives, we observed challenges to identifying language appropriate to explain biobanking and genetic testing to our target population. Engagement with communities to adopt contextually relevant terminologies that participants can understand is crucial. Researchers need to be mindful of the impact of communities’ socio-economic status and how compensation can be potentially coercive.

## Introduction

The concern that potential coercion could undermine the validity of informed consent is a key ethical challenge to clinical research in lower- and middle-income countries (LMICs). Some researchers have reported concerns of coercion due to unequal power dynamics and socio-economic disadvantage.^1^^–^^3^ These vulnerabilities have led to debates on the validity of the informed consent process questioning the competence, comprehension and voluntariness of research participants in these contexts.^4^^,^^5^ This is especially true of research with complex procedures and involving innovative approaches such as genetics and biobanking.^6^^–^^8^ Biobanking involves the collection of biological samples, including blood, urine and other human body tissues and fluids. These approaches have the potential to transform healthcare, leading to precision medicine that involves knowing an individual's genomic traits, thus making it easier to guide disease prevention, diagnosis and treatment.

However, more ethical dilemmas are introduced with these innovative approaches, including how researchers explain the magnitude and international nature of data sharing that is part of biobanking and how they guarantee the confidentiality and protection of participants when their biological samples are stored for future research use.^9^ Biobanking is essential to support research that will identify genetic and environmental factors contributing to diseases in Africa and improve the health of the African population. However, a question of whether research participants understand the magnitude of their consent remains unanswered.

Since 2000, the Africa Health Research Institute (AHRI) has maintained a longitudinal population-based health and demographic surveillance system (HDSS) in rural KwaZulu-Natal, South Africa, with optional human immunodeficiency virus (HIV) testing added in 2003. The area is 854 km^2^ in size and includes a population of approximately 90 000 residents (people who spend most of their nights in the surveillance area) and 40 000 non-residents (people who are part of the households but physically spend most of their nights outside the surveillance area) who are members of approximately 20 000 households. In 2018, in response to the epidemiological shift and an increased focus on biomedical science, the AHRI established a multimorbidity cohort and accompanying specimen biobank. Roving community-based health camps for multidisease screening (HIV, tuberculosis, hypertension and diabetes) and the collection of biosamples were deployed throughout the HDSS.^10^ The goal of the program was to assess pathogen, host and environmental determinants of health and disease, leading to the development of novel scientific and public health interventions. The programme is known as Vukuzazi (‘wake up and know ourselves’) in the local language (isiZulu). Explaining the Vukuzazi research objectives was an essential component of engagement with participants, the wider local research community and stakeholders to ensure the ethical conduct of research that met community needs.^2^^,^^11^^,^^12^

In this article we describe the contextually informed experiences and perceptions of the research objectives by participants in Vukuzazi.

## Methods

### Study setting

The study was conducted in the uMkhanyakude district, KwaZulu-Natal, which is the second most deprived district in South Africa, with a high incidence and prevalence of HIV.^13^^,^^14^ In this predominantly rural area, there are high levels of poverty and unemployment and the proportion of individuals with no formal education is higher than that of the national population.^13^

### Vukuzazi process of community engagement

The study was first presented and discussed with the AHRI Community Advisory Board (CAB) and other local stakeholders, including elected and traditional leaders and the Department of Health. Discussions included aspects such as the request for self-administered rectal swabs, the process of collecting biological samples and some limitations of AHRI, such as in cancer screening, which the community members were interested in. This was followed by presentations through roadshows to the community by the AHRI public engagement team. At these roadshows, details of several AHRI projects were discussed, giving community members the opportunity to ask questions and engage with the institute's research. At several key time points (after the pilot, after enrolment of the first 1000 participants, after 6 and 12 months) the study team met with the CAB to give feedback on the community's experience of the study and to present preliminary results.

### Vukuzazi recruitment and informed consent process

Local isiZulu-speaking fieldworkers (individuals with a senior school certificate [grade 12] and at least 2 years of experience in a clinical research setting) visited eligible participants in their homesteads to invite them to participate. During this visit, potential participants received a verbal explanation of the study and a pictorial brochure in isiZulu that summarized the research objectives, activities, risks/benefits and reimbursement (see supplementary material). Eligible participants were given at least 1 day to read and consider the materials and decide whether to come to the health camp. In homes with eligible minors (15–17 years of age), the fieldworkers first engaged the parents/guardian and upon obtaining parental/guardian approval, approached the minor with study materials and an invitation to attend the health camp.

Upon arrival at the health camp, potential participants received a 14-page written informed consent information sheet in isiZulu that they could read while waiting (see supplementary materials). Using an electronic system to document the process, a clinical research assistant trained in Good Clinical Practice conducted a private informed consent process using a script and pictorial aids that had been refined based on participant and staff feedback after the pilot phase. Participants had the opportunity to ask questions and the professional nurses were also available to respond to any queries. Consent was obtained from adult participants and assent from minors whose parent/guardian had provided consent. Prior to biosample collection, a study nurse explained disease screening and referral algorithms and reconfirmed consent to be screened for each condition and receive clinical referrals based on their results. At this stage participants could selectively withdraw their consent from any particular study procedure without losing access to all other study components. After their visit to the health camp, participants received their results by short message service (if normal) or during a one-on-one home visit by a primary healthcare nurse who explained their tests results, performed confirmatory measurements or tests and made referrals to the public health system for treatment.

### Qualitative substudy sample

For this substudy, a random sample of participants and non-participants was identified by the statistician using a computer algorithm stratified by sex, age, participants, non-participants and different states of health and disease. Staff members, representing different positions in the team, were also sampled for interview. Prior to conducting the interviews, the qualitative study was explained and informed consent for audio-recorded interviews was obtained.

### Qualitative substudy data collection

Semi-structured interviews were conducted by a social science research assistant in the interviewee’s preferred language (isiZulu or English) and location. The topic guide covered reasons for participation or non-participation, the recruitment process, the interviewee's understanding and perception of study procedures (including ethical aspects, especially informed consent and storage and future use of biological samples). Staff members were asked about their knowledge and understanding of participants’ understanding of the study and motivation to participate (see supplementary material).

### Qualitative substudy data management and analysis

The interviews were digitally recorded, audio files were transcribed and those conducted in isiZulu were translated into English. All identifying information was removed from the interview transcripts. The transcripts and emerging findings were reviewed and discussed by the social science team. From these discussions, a coding framework was developed based on the themes from the topic guide and themes emerging from the data. The transcripts were coded manually and analysed thematically.

### Ethical approval

Ethical approval for Vukuzazi and the qualitative substudy was obtained from the University of KwaZulu-Natal Biomedical Research Ethics Board (BE560/17), the London School of Hygiene and Tropical Medicine Ethics Committee (14722) and the Partners Institutional Review Board (2018P001802).

## Results

Thirty-nine individuals took part in the substudy: 31 invited participants (24 participants, 7 non-participants) and 8 members of the research team (representative of different study staff cadres: fieldworker, driver, nurse, investigator). Table 1 shows the sampling frame used to recruit.

**Table 1.** Sample selection criteria

Gender	Sample			
Males	Three non-participants (one <24 y, one 24–50 y, one >50 y)	Three with either new active TB or new HIV (one <24 y, one 24–50 y, one >50 y)	Five with multimorbidity (i.e. with two of either HIV, active or prior TB, diabetes or hypertension) (one <24 y, one 24–35 y, one 36–50 y, two >50 y)	Four with no disease (one <18 y, one 18–24 y, one 25–45 y, one >45 y)
Females	Three non-participants (one <24 y, one 24–50 y, one >50 y)	Three with either new active TB or new HIV (one <24 y, one 24–50 y, one >50 y)	Five with multimorbidity (i.e. with two of either HIV, active or prior TB, diabetes or hypertension) (one <24 y, one 24–35 y, one 36–50 y, two >50 y)	Four with no disease (one <18 y, one 18–24 y, one 25–45 y, one >45 y)

## Participant responses

### Participant understanding of the informed consent process

The majority of participants reported that they understood the study procedures and had taken the time to read through the information sheet, as reported by a 33-year-old man:

*It was all in the paper they gave us, I took my time to read and understand it. It was quite thick. It was asking us to consent to participate and it was talking about how certain illnesses are contracted and what not.*

Participants with limited literacy indicated that verbal explanations by the study staff allowed them to understand their rights as a research participant despite their inability to read the written materials, as described by a 54-year-old woman:

*I understood that nothing was compulsory so if you didn't want to do something you could just say so. I signed with a cross because I don't know how to read*.

### Participants’ understanding of the research objectives

Most participants understood that Vukuzazi sought to determine the patterns of health and disease in their community. For some participants, understanding of this objective was difficult to fully distinguish from the perceived healthcare delivery components of the platform. A 22-year-old man said:

*They explained. They said they want to see how the community is doing health-wise after five years. That's what they were doing. They want to see how healthy they will be. Maybe some of those people would have died after five years if AHRI hadn't come to help us. Some people found out they were sick, and they were linked to the treatment so they will live for five years* *—* *I know a few of them.*

### Genetics and biobanking

When asked about biobanking and the genetic aspects of the research, participants could not remember what they were told during the informed consent process. After the researcher explained biobanking, some participants expressed positive views, as a 28-year-old woman shared when asked about her feeling on the storage of samples collected:

*I don't think there's anything negative about it. I think it is good because it will help us at the end of the day.*

On the other hand, it seemed that others did not understand biobanking or the process of storing samples and thought that it was a bad idea, as this 24-year-old man shared:

*It's a bad idea. It must be stopped…No man, storing a human being's blood in the fridge? Where have you ever seen someone staying in a fridge? No, it's a bad idea. Beer stays in the fridge, not human blood.*

Most participants said genetics and biobanking were complex and therefore not easy to remember. A 28-year-old woman offered advice when asked how we can improve knowledge about genetics research in the community:

*You can explain it by saying that the research looks at hereditary characteristics in your family and how they relate to illness that one has or might be susceptible to. It's not necessarily that you got infected….Yes, the ‘hereditary’ concept needs to be emphasised*.

### Reasons for participation: free convenient health check

Most participants stated that the main reason for their participation was the benefit of receiving a free health check and potentially receiving healthcare services. The location of the mobile health camp close to their homestead provided a convenient means to access healthcare, as a 67-year-old woman stated:

*I've always been someone who likes going for regular check-ups. I like knowing where I stand with all the many diseases that exist nowadays. I like knowing what my situation is and I figured it might be better to go to Vukuzazi because it had located itself so close and convenient to us.*

For some, the convenience and affordability of the service offered at the health camp motivated them to participate; attendance meant incurring no travel or medical costs with minimal waiting time. A 36-year-old woman compared participation in Vukuzazi with the costs she would incur if she had the same tests in a health facility:

*I was talking to a friend of mine about how expensive all the things we were doing there [at the camp] are if you do them with your own money or medical aid. Hospitals charge a lot of money for those procedures.*

### Reasons for participation: loyalty to AHRI

Some participants indicated that they had been part of AHRI research projects for a long time and therefore did not see any reason not to continue with their participation. A 75-year-old woman explained:

*[T]* *hey [AHRI] have been coming to us for a long time now. So why would we stop taking part now when we have been doing so for such a long time? Girls would visit us to sign us up and we've had no problem, so it was the same thing this time around.*

A 41-year-old man said he wanted to continue supporting the institute:

*We have been supporting AHRI for a long time now, from back when it was Africa Centre and my grandmother was still alive. We are still supporting them now with Vukuzazi and we will continue to do so.*

### Reasons for participation: reimbursement as an incentive

Participants in Vukuzazi received reimbursement in the form of a food voucher (100R [US$6]) and snack pack (water, apple, biscuits), in accordance with South African National Health Research Ethics Committee guidelines, which recommend consideration of the participant's time, inconvenience and expenses.^15^ Outside of the reimbursement calculation, participants also received a Vukuzazi T-shirt that they changed into for the portable chest X-ray procedure and then were allowed to keep. Some participants appreciated the reimbursement, including the food, they were given, which strengthened their loyalty to the AHRI. A 64-year-old man stated:

*What I really liked most is that AHRI gave me food and R100 ($6). I was able to send a child to go buy a few things for me. This made me realise that there's no way we could chase AHRI away like we would do with before [in the past when the Institute focused on HIV] because they are on our side and they care about us so much that they can give us food.*

Others shared how they thought some members of the community were not interested in the study objectives and wanted the voucher or to receive something, as a 38-year-old woman stated:

*They just go there because they heard they will get something in return. Not because they genuinely want to wake-up and know themselves like the name of the study says.*

### Reasons for non-participation

Some of the people contacted said that they were unable to participate because they had other commitments at the time when Vukuzazi was in their local area or because Vukuzazi operated primarily during normal working hours, when they were at work.

*I was working day shift that whole week, otherwise I would've gladly taken part.* — 23-year-old man

 

*Oh, I was away because of work. They came here to invite me but I was not home but they left my invitation with my mom and she called me to tell me about it.* — 52-year-old man

However, some participants speculated about reasons that their peers may have chosen not to participate in the programme. The rate of non-participation was highest among young men and when asked about this a 22-year-old male participant suggested:

*It is very hard for men to ask for or accept any sort of help. With others it's just a matter of ignorance, they aren't very clued up about a lot of things* *—* *health being one of them, hence they don't see the value in a project like Vukuzazi.*

One person did not participate in Vukuzazi because he felt that the AHRI kept taking information from the community without giving back aid to address the poverty they were facing.

## Research staff responses

Some research staff thought participants may have participated because of the financial reimbursement and other benefits. The staff said that such people had not appeared to be interested in the explanation of the study being given during the informed consent process. One staff member recalled:

*There are people that you explain everything to, but you can see that they aren't really interested because they are just there for the voucher. After taking blood samples we ask people if they would like to receive their results and some participants say ‘no, you can give me my diabetes results but I would not like to receive my HIV results.’ So, you can see that person isn't really there for his health, it's just the voucher.*

When it came to aspects of biospecimens and storage of samples, few participants seemed to understand the information that had been given to them. One explanation for this could be the way the recruiters may have described these concepts. As a member of the research team explained:

*What I can say about genetic testing is that we don't tell them too much about this. What we tell them is that there is a blood sample that we are going to use to test their genetics and they know that they won't get the results of these tests because the research is still under way. Some people don't really understand what genes are even when we explain it to them.*

## Discussion

We found that most participants believed that they received adequate information to decide on participating in the health screening programme. However, others reported not understanding the content completely but signed the consent form as an indication of voluntariness. It is possible that true voluntariness may have been compromised by some participants’ focus on the reimbursement or the perceived medical benefits of participating. The latter may have led participants to not fully comprehend the difference between Vukuzazi as a research programme and as a healthcare service providing convenient access to good quality care. This is consistent with previous empirical evidence indicating that research participants in LMICs can have a high propensity to therapeutic misconceptions and have an expectation of healthcare from research.^16^^–^^19^ It is not possible to ignore the socio-economic factors that may promote research participation in situations of poverty with limited access to resources and healthcare services.^20^^,^^21^ This vulnerability plays a role in voluntariness and the value placed on reimbursements or access to healthcare that exceeds local standards.^22^ Our results illustrate these challenges faced by participants and the scientific community as they strive for ethical inclusion of vulnerable populations into research studies.

The subject of participant reimbursement or compensation is contentious in LMICs due to the potential undue influence it can have on participants. Addressing this issue can be challenging and engaging with the community through the CAB on understanding and developing a culturally sensitive method of compensation is highly advised, as well as transparency by informing participants whether they will be compensated or not and an explanation of what the compensation is for. This will help minimise the perception of viewing this as a benefit and maintain scientific integrity.

We found that understanding of the concept of ‘future use’ of biosamples for genetic research was quite limited. Participants struggled to recall the genetics research information that had been shared during the informed consent process, although when the interviewer explained these concepts, most had positive views and thought biobanking was a good thing. The lack of understanding of biobanking and genetic research is similar to previous research in other settings where participants had difficulty in comprehending such concepts.^23^^,^^24^ This highlights the need for strategies such as better explanations of these complex concepts, working with communication professionals in developing material, cognitive testing to check comprehension and involving native speakers with knowledge of the study area.^25^^–^^28^ One participant suggested focusing on terminology that is easily understood by the community, such as ‘hereditary characteristics’ in explaining genetics research. This is in line with previous research and has been identified as a framework tenet for informed consent of genetic research in Africa.^8^

This study highlighted how some forms of structural coercion may exist whereby community members are loyal to a research institute, which may influence power dynamics and voluntariness.^29^ Researchers need to anticipate these structural and power issues that may be in play within such communities and develop innovative ways of checking and ensuring that agreement to participate is given freely.

Despite these multiple barriers to comprehension and ethical challenges experienced, many participants affirmed that they understood that the purpose of the research was to better understand the nature of health and disease in the community. Even though some of these interviews were conducted months after participants engaged with the health camp, they recalled the rationale for some of the procedures and were able to share their understanding. This indicated an enduring and internalized comprehension of at least some of the sophisticated research objectives by most participants.

One strength of this study is that we were able to triangulate data sources, which was helpful in shedding light and identifying the potential mismatch in information between what researchers believe participants understood and what the participants themselves express. A limitation is that we failed to collect sufficient data on participants’ understanding of the genetic and biobanking aspects of the study. Our interviewer had difficulty engaging participants about both these themes. While this could reflect the complex nature of these concepts and terminologies used, it might be reflective of the duration between the study and the interviews or a failure to adequately explain these procedures during the consenting process. As genomic and biobanking studies are expanding in both scope and number, future work should better explore study participant understanding of these issues in human subjects’ research.^7^

## Conclusions

The informed consent process in clinical research in LMICs is often challenging and requires careful preparation, especially in settings where there has been a long history of research and in which open-ended biobanking protocols are being introduced. It is possible that people take part in research and the procedures they are asked to undertake on trust, because of the long-standing relationship, without engaging with the purpose of new studies. In a context where there are high rates of unemployment, poverty and low literacy, care is required to both ensure the purpose of the research is explained clearly and any suggestion of research procedures being misconstrued as a free health service addressed. Our findings underline the importance of engagement of the community to ensure that the research objectives serve the needs of the community, engaging in thoughtful development of consent forms that are appropriately targeted in language and delivery method to the study population, following up with participants to assess understanding and answer questions about the research they take part in, and the importance of training research staff to give time and care to the process of guiding a participant through the informed consent process.

## Supplementary data

Supplementary material are available at International Health online.

## Supplementary Material

ihaa072_Supplemental_File

## Data Availability

Data cannot be shared publicly because of confidentiality and potential breach as the data contains potentially identifying participant information. Data are available from the AHRI Research Data Management committee (contact via RDMServiceDesk@ahri.org) for researchers who meet the criteria for access to confidential data.
