# Supplementary material for: Participant understanding of informed consent in a multidisease community-based health screening and biobank platform in rural South Africa
Source: Int Health. 2020 Nov 9;12(6):560–6. doi: 10.1093/inthealth/ihaa072 (PMC7651191; doi:10.1093/inthealth/ihaa072)
Supplement: ihaa072_Supplemental_File [file ihaa072_supplemental_file.zip › Topic_guide_for_Research_Team.docx]

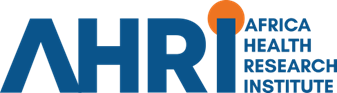


Research Team Interview Schedule

- Let’s start with you telling me a little bit about yourself (where you’re from, when you started working here at AHRI and what you do here)
- Tell me what you understand the Vukuzazi study to be all about (probe for why certain procedures are done, why is AHRI even doing Vukuzazi)
- What made you want to be a part of this study?
- How are participants recruited onto this study? (Probe for how the study is explained to Participants)
- What role do the participants play? (probe for what happens at every step, i.e testing, sample collection, storage etc)
- What is your role in this study?
- Apart from yours, what other roles are performed in this study?
- What ethical procedures took place during this study? (Probe for ethical challenges/dilemmas)
- In general, have there been any unexpected challenges or hiccups so far?
- What do you enjoy the most about your role?
- What do you enjoy the least?
- Is there anything you think could be improved in this study?
