# Supplementary material for: Participant understanding of informed consent in a multidisease community-based health screening and biobank platform in rural South Africa
Source: Int Health. 2020 Nov 9;12(6):560–6. doi: 10.1093/inthealth/ihaa072 (PMC7651191; doi:10.1093/inthealth/ihaa072)

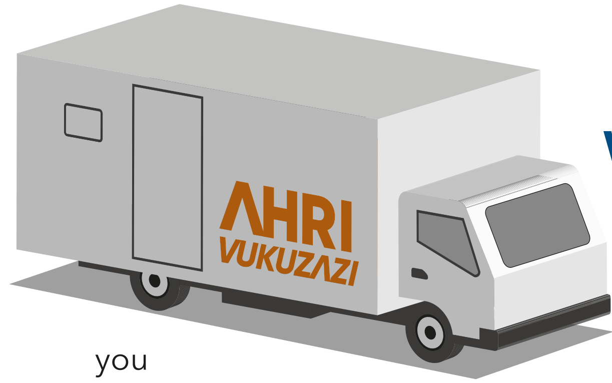

What to expect

AHRI Clinic Research Assistants will visit you at your home and ask if you are interested in volunteering to be part of Vukuzazi. If you are, they will point you towards the mobile clinic and mobile X-ray vehicles which will be parked in a central area in your neighbourhood. Everyone over the age of 15 who lives in our study area will have the opportunity to participate in the research – if you would like to. The health screenings will take place very close to your home and will require 2-3 hours of your time on the day of the screening. We want you to be comfortable during the time you spend at the health screening, so we will provide a shaded place to sit while you wait, and food and water for you to drink and eat after you have finished your testing. Because your time is valuable and the health screening will involve some inconvenience, we will also provide a food voucher to reimburse and thank all participants for their time.

Many diseases start off without symptoms – if we catch these diseases early by doing health screenings, we can help to prevent them becoming more serious. If you choose to participate in the health screening, you will be asked questions about your health and lifestyle. We will collect bloods in order to screen you for diabetes and HIV. We will measure your blood pressure, weigh you and do chest X-rays to look for TB. All of these discussions and tests will be done in a private space and your information and results will be kept confidential. You will need to change into a T-shirt for the chest X-ray and you will be able to keep this T-shirt when you go home. If any of these screening tests show signs of health problems, we will return to your home in a few weeks to re-check your results and help you to get treatment in the clinic if you need it. If all your results come back showing that you are healthy, we will provide you with these results by SMS.

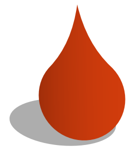

In addition to a blood sample, we will also be collecting rectal swab samples. This is because the kind of bacteria (good and bad germs) that live in our body can also play a role in how healthy or sick we are. These germs can be identified in our stool or on a rectal swab. We want to study how these germs may influence health and disease in our community. This is a new area of science called 'microbiomics'. You can collect the rectal swab yourself in a mobile toilet or a nurse can help you with this.

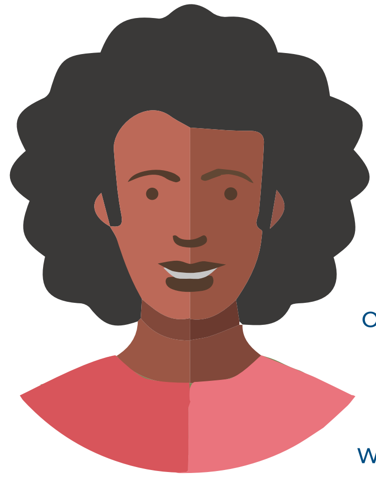

We will also use your blood sample to study something called 'genes'. These genes are present in all of us and are responsible for why people in families look like each other. This kind of information is passed from parents to their children and helps to decide, for example, how tall you will be and what your body shape will be. Some of these genes may prevent us from getting sick and some other genes may be one of the reasons we get sick when others do not. Studying genes along with health information will help us better understand what causes certain diseases in our community.

Why does AHRI want to do genetic testing?

Over the past 10 years, technology called 'next-generation genetic sequencing' has allowed scientists to get new insights into human health and disease. This has created the possibility of new and better personalised treatments for diseases, based on a person's genetics (what you inherit from your parents). Despite Africa having the greatest genetic diversity on the planet, there has been very little genetic research done on the continent. Vukuzazi gives us a chance to focus the power of genetics on the health problems that are relevant to our community.

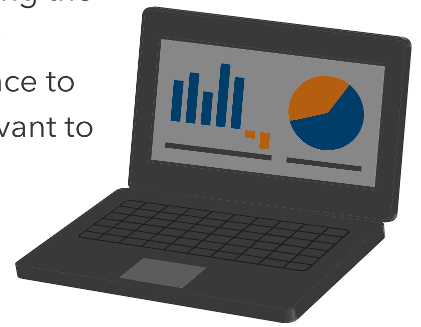

Why is Vukuzazi important?

AHRI hopes to get a better understanding of rates of health and disease in the population and how these diseases interact with each other. We want to figure out the best ways to screen people for diseases and to get them onto effective treatments. We also want to use modern technology to tackle the diseases that are most affecting our population and discover new ways to prevent or better treat these diseases.

If you have questions please contact the Ethics Committee:
Biomedical Research Ethics Committee
Research Office, Govan Mbeki Centre, Westville Campus, University of KwaZulu-Natal
Fax: 031 260 4769 | e-mail: BREC@ukzn.ac.za
BREC Reference number: BE560/17

AHRI AFRICA HEALTH RESEARCH INSTITUTE
719 Umbilo Road, Durban
R618 to Hlabisa, Somkhele, KwaZulu-Natal
031 260 4955 I 035 550 7506

AHRI VUKUZAZI

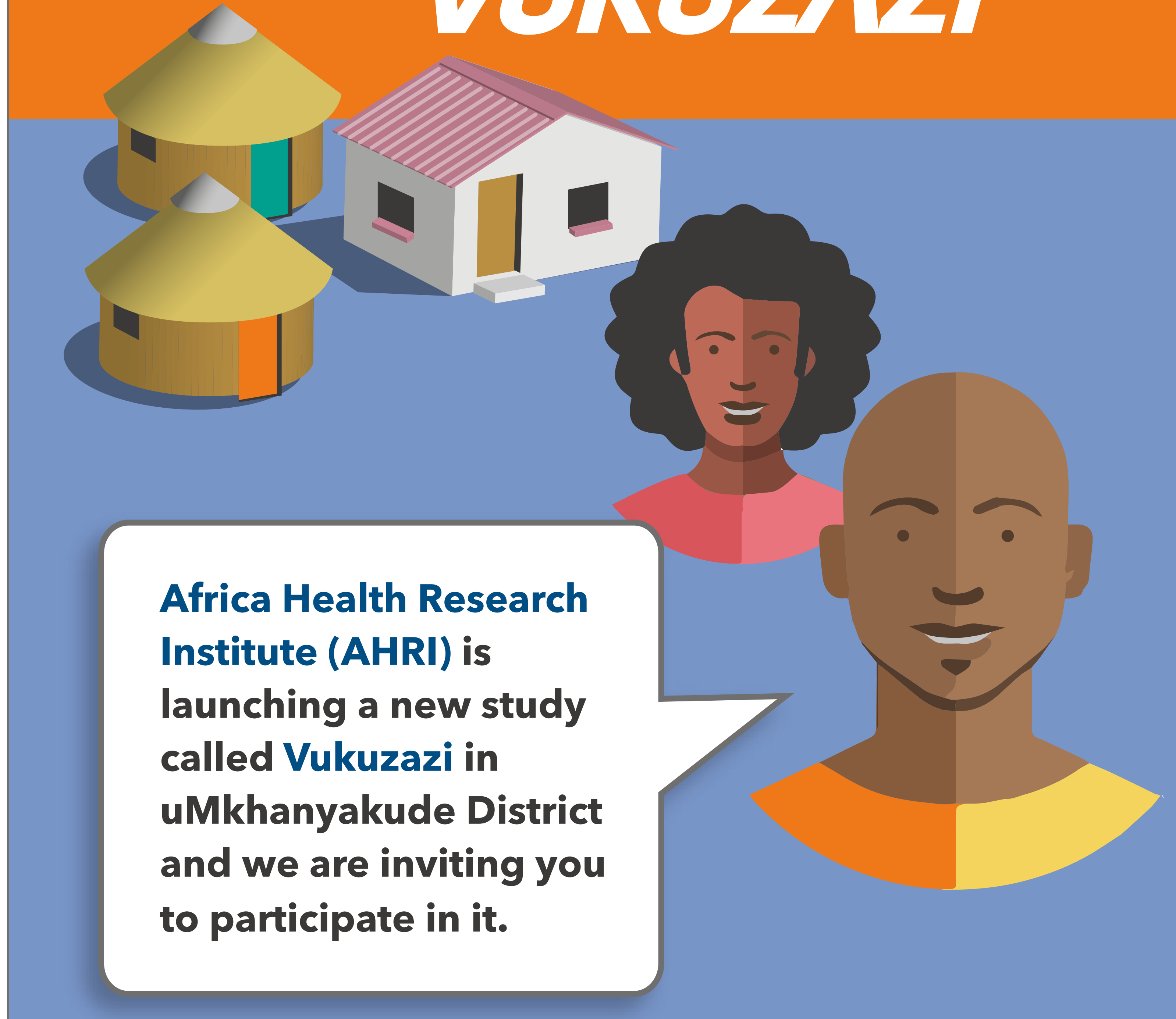

Africa Health Research Institute (AHRI) is launching a new study called Vukuzazi in uMkhanyakude District and we are inviting you to participate in it.

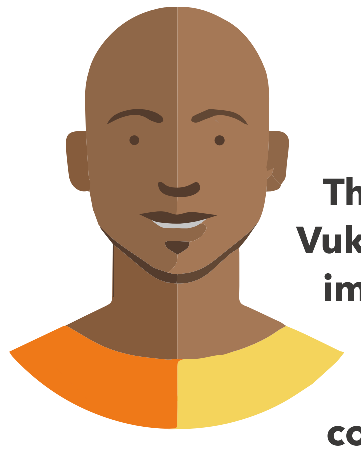

The isiZulu word vuk'uzazi refers to 'knowing ourselves'. Vukuzazi will involve health screenings for the most important diseases that affect our community. Through this health screening we will come to understand a lot more about the state of health and disease in our community and in South Africa as a whole. For example, we would like to know how common diabetes and high blood pressure are and how they interact with HIV and tuberculosis (TB). We also want to learn how to diagnose these health problems better and to understand why different people's bodies have different responses to infections. Using the information we collect in Vukuzazi, we can help to plan future health care to prevent and treat diseases affecting our community.

'Knowing ourselves' is the first step in improving the health of individuals and the wider population, which is the ultimate goal of Vukuzazi

We will be inviting about 50 000 people older than 15 from across uMkhanyakude District to take part in the study. Vukuzazi screenings will start in May 2018 and will finish at the end of 2019. Our research on the samples and information that we gather will continue for many years. We are working in partnership with the Department of Health to ensure quick and effective treatment of diseases which we diagnose.

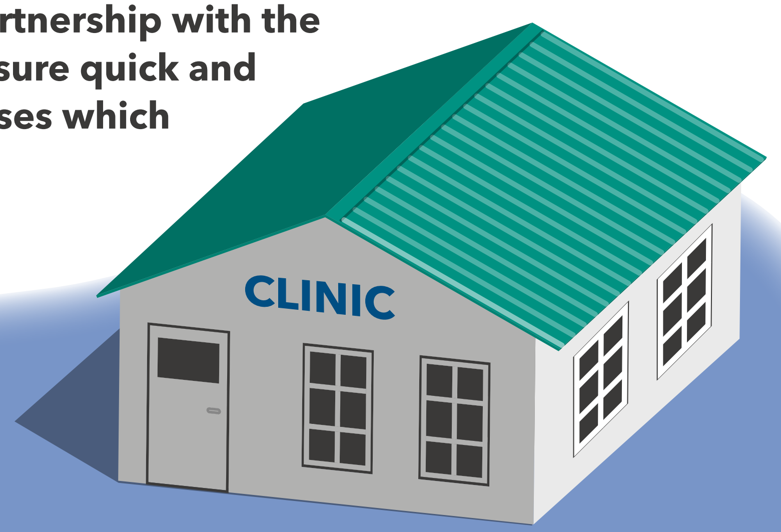

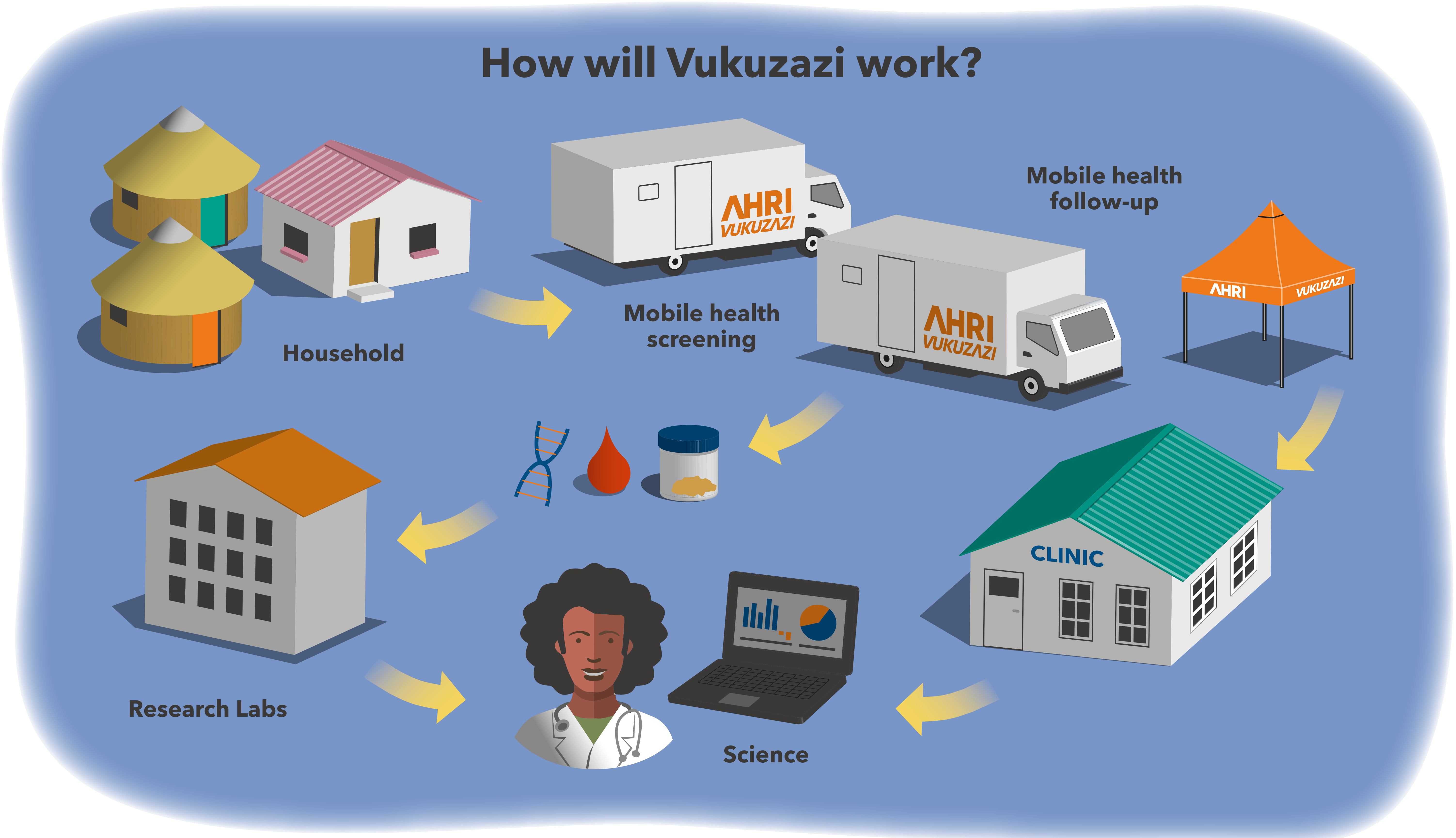

Supplement: ihaa072_Supplemental_File [file ihaa072_supplemental_file.zip › Vukuzazi_Pamphlet_English_10_05.pdf]
