# Supplementary material for: Participant understanding of informed consent in a multidisease community-based health screening and biobank platform in rural South Africa
Source: Int Health. 2020 Nov 9;12(6):560–6. doi: 10.1093/inthealth/ihaa072 (PMC7651191; doi:10.1093/inthealth/ihaa072)
Supplement: ihaa072_Supplemental_File [file ihaa072_supplemental_file.zip › Vukuzazi_team.docx]

**Vukuzazi Team - Below is a list of staff that significantly contributed to the implementation and conduct of Vukuzazi.**

***** Vukuzazi Study Group. The study group members were closely involved with the design, implementation and oversight of Vukuzazi (listed in pubmed).

| **Name** | **Role** |
| --- | --- |
| *Deenan Pillay | Principal Investigator (2017-2019) |
| *Willem Hanekom | Principal Investigator (2019-present) |
| *Emily Wong | Co-Principal Investigator |
| *Mark Siedner | Co-Principal Investigator |
| *Olivier Koole | Co-Principal Investigator (2017-2019) |
| *Thumbi Ndung’u | Co-investigator |
| *Thandeka Khoza | Co-investigator (2019-present) |
| *Kobus Herbst | Co-investigator |
| *Kathy Baisley | Co-investigator |
| *Janet Seeley | Co-investigator |
| *Alison Grant | Co-investigator |
| *Resign Gunda | Programme Manager |
| *Ashmika Surujdeen | Study Coordinator |
| *Theresa Smit | Head: Diagnostic Research |
| *Dickman Gareta | Head: Research Data Management |
| *Day Munatsi | Head: Research Data Systems |
| *Ngcebo Mhlongo | Study Physician |
| *Sanah Bucibo | Lead Nurse |
| *Nompilo Buthelezi | Training Coordinator |
| *Tshwaraganang Modise | Research Data Manager |
| *Jaco Dreyer | Senior Research Data Manager |
| *Siyabonga Nxumalo | Research Data Manager |
| *Stephen Olivier | Statistician |
| *Innocentia Mpofana | Diagnostic Laboratory Manager |
| *Gregory Ording-Jespersen | Laboratory Data Supervisor |
| *Khadija Khan | Biorepository Manager |
| *Zizile Sikhosana | Somkhele Laboratory Supervisor |
| *Sashen Moodley | Microbiology Laboratory Supervisor |
| *Hollis Shen | Head: Exploratory Research Division |
| Kennedy Nyamande | Pulmonology Consultant |
| Jaikrishna Kalideen | Radiologist |
| Ramesh Jackpersad | Radiologist |
| Kgaugelo Moropane | Radiographer |
| Boitsholo Mfolo | Radiographer |
| Khabonina Malomane | Radiographer |
| Hlolisile Khumalo | Nursing Manager |
| Nozipho Mbonambi | Professional Nurse |
| Hloniphile Ngubane | Professional Nurse |
| Thokozani Simelane | Professional Nurse |
| Khanyisani Buthelezi | Professional Nurse |
| Sphiwe Ntuli | Professional Nurse |
| Nombuyiselo Zondi | Professional Nurse |
| Siboniso Nene | Professional Nurse |
| Bongumenzi Ndlovu | Enrolled Nurse |
| Talente Ntimbane | Enrolled Nurse |
| Mbali Mbuyisa | Enrolled Nurse |
| Xolani Mkhize | Enrolled Nurse |
| Melusi Sibiya | Enrolled Nurse |
| Ntombiyenkosi Ntombela | Enrolled Nurse |
| Mandisi Dlamini | Enrolled Nurse |
| Hlobisile Chonco | Enrolled Nurse |
| Hlengiwe Dlamini | Enrolled Nurse |
| Doctar Mlambo | Enrolled Nurse |
| Nonhlanhla Mzimela | Enrolled Nurse |
| Zinhle Buthelezi | Enrolled Nurse |
| Zinhle Mthembu | Enrolled Nurse |
| Thokozani Bhengu | Enrolled Nurse |
| Sandile Mthembu | Enrolled Nurse |
| Phumelele Mthethwa | Enrolled Nurse |
| Zamashandu Mbatha | Enrolled Nurse |
| Welcome Petros Mthembu | Enrolled Nurse |
| Anele Mkhwanazi | Clinical Research Assistant Supervisor |
| Mandlakayise Zikhali | Clinical Research Assistant Supervisor |
| Phakamani Mkhwanazi | Clinical Research Assistant |
| Ntombiyenhlanhla Mkhwanazi | Clinical Research Assistant |
| Rose Myeni | Clinical Research Assistant |
| Fezeka Mfeka | Clinical Research Assistant |
| Hlobisile Gumede | Clinical Research Assistant |
| Nonceba Mfeka | Clinical Research Assistant |
| Ayanda Zungu | Clinical Research Assistant |
| Hlobisile Gumede | Clinical Research assistant |
| Nonhlanhla Mfekayi | Clinical Research Assistant |
| Smangaliso Zulu | Clinical Research Assistant |
| Mzamo Buthelezi | Clinical Research Assistant |
| Senzeni Mkhwanazi | Clinical Research Assistant |
| Mlungisi Dube | Clinical Research Assistant |
| Philippa Mathews | Clinical Governance |
| Hosea Kambonde | IT Systems Developer |
| Lindani Mthembu | Information Technology Assistant |
| Seneme Mchunu | Information Technology Assistant |
| Sibahle Gumbi | Research Admin Assistant |
| Tumi Madolo | Research Data Manager |
| Thengokwakhe Nkosi | Driver |
| Sibusiso Mkhwanazi | Driver |
| Sibusiso Nsibande | Driver |
| Mpumelelo Steto | Driver |
| Sibusiso Mhlongo | Driver |
| Velile Vellem | Driver |
| Pfarelo Tshivase | Driver |
| Jabu Kwinda | Driver |
| Bongani Magwaza | General Worker |
| Siyabonga Nsibande | General Worker |
| Skhumbuzo Mthombeni | General Worker |
| Sphiwe Clement Mthembu | General Worker |
| Antony Rapulana | Laboratory Technologist |
| Jade Cousins | Laboratory Technologist |
| Thabile Zondi | Laboratory Technologist |
| Nagavelli Padayachi | Laboratory Technologist |
| Freddy Mabetlela | Laboratory Technologist |
| Simphiwe Ntshangase | Laboratory Technician/LIMS Administrator |
| Nomfundo Luthuli | Laboratory Technician |
| Sithembile Ngcobo | Laboratory Technologist |
| Kayleen Brien | Laboratory Technologist |
| Sizwe Ndlela | Laboratory Technician |
| Nomfundo Ngema | Laboratory Technician |
| Nokukhanya Ntshakala | Laboratory Technician |
| Anupa Singh | Laboratory Technician |
| Rochelle Singh | Laboratory Technician |
| Logan Pillay | Laboratory Technician |
| Kandaseelan Chetty | Laboratory Technician |
| Ashentha Govender | Laboratory Technician |
| Pamela Ramkalawon | Laboratory Research Technician |
| Nondumiso Mabaso | Laboratory Intern |
| Kimeshree Perumal | Laboratory Intern |
| Senamile Makhari | Biorepository Laboratory Technician |
| Nondumiso Khuluse | Biorepository Laboratory Technician |
| Nondumiso Zitha | Biorepository Research Assistant |
| Hlengiwe Khathi | Biorepository Research Assistant |
| Mbuti Mofokeng | Clinical Specimen Driver/Laboratory Assistant |
| Nomathamsanqa Majozi | Public Engagement |
| Nceba Gqaleni | Public Engagement |
| Hannah Keal | Communications |
| Phumla Ngcobo | Communications |
| Costa Criticos | Operational Oversight |
| Raynold Zondo | Operational Oversight |
| Dilip Kalyan | Operational Oversight |
| Clive Mavimbela | Operational Oversight |
| Anand Ramnanan | Procurement |
| Sashin Harilall | Grants Office |
