# Supplementary material for: Participant understanding of informed consent in a multidisease community-based health screening and biobank platform in rural South Africa
Source: Int Health. 2020 Nov 9;12(6):560–6. doi: 10.1093/inthealth/ihaa072 (PMC7651191; doi:10.1093/inthealth/ihaa072)
Supplement: ihaa072_Supplemental_File [file ihaa072_supplemental_file.zip › Topic_guide_for_participants.docx]

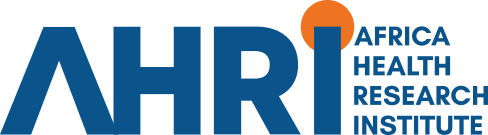


**Follow-up Study on Vukuzazi Experience**

Interview Schedule for Participant Interviews

- Tell me about how you were recruited onto Vukuzazi?
- What were you told the study is about? (Probe for understanding of the study, i.e why AHRI is doing it, informed consent, storage and use of samples)
- What kind of people do you think this study was designed to benefit? (probe for whether they’d encourage others to participate and why)
- Let’s talk about why you decided to participate (Probe for perceived benefits of participation, probe for why some people might opt not to participate, probe for why a small number of men participated)
- Talk to me about how you found the entire experience (probe for likes and dislikes of participation, thoughts on the various procedures, parts enjoyed the most, parts enjoyed the least)
- Having participated and received your results, how do you reckon you’ve benefited from this study? (probe for what they think happens after their samples have been collected)
- What do you think might have made other people decide not to participate?
- Is there anything you would change about the design of the project and/or your experience?
- Would you encourage other people to participate? Why?
